# CD8-positive memory T cells in tumor-draining lymph nodes of patients with breast cancer

**DOI:** 10.1186/s12885-020-6714-x

**Published:** 2020-03-30

**Authors:** Yasmin Vahidi, Mandana Bagheri, Abbas Ghaderi, Zahra Faghih

**Affiliations:** 1 Shiraz Institute for Cancer Research, School of Medicine, Shiraz University of Medical Sciences, P.O. Box: 71345-1798, Shiraz, Iran; 2 Department of Pathology, Shiraz Central Hospital, Shiraz, Iran

**Keywords:** Breast cancer, Lymph node, CD8^+^ memory subsets, Memory stem cells

## Abstract

**Background:**

Human immunological memory is a hallmark of the adaptive immune system and plays an important role in the development of effective immune responses against tumors. In the present study, we aimed to determine the frequencies of CD8^+^ memory T cell subsets including T stem cell memory (TSCM) in tumor-draining lymph nodes of patients with breast cancer (BC).

**Methods:**

Mononuclear cells were obtained from axillary lymph nodes of 52 untreated patients with BC and stained for CD8, CCR7, CD45RO, CD95 markers to detect different subtypes of memory cells in the CD8^+^ lymphocyte population. Data were acquired on four-color flow cytometer and analyzed with CellQuest Pro software.

**Results:**

We observed that 47.65 ± 2.66% of CD8^+^ lymphocytes expressed the CD45RO, a marker for memory T cells. Statistical analysis showed that the total frequency of central memory T cells (TCM) and their subset with low CD45RO expression was significantly higher in tumor-involved nodes compared to tumor-free ones (*P* = 0.024 and *P* = 0.017, respectively). The level of CD95 expression (based on mean fluorescence intensity) on the surface of TCM, their CD45RO^hi^ and CD45RO^low^ subsets, and TSCM was higher in patients with stage II compared to those in stage I (*P* < 0.05). In addition, the percentage of naive CD8^+^ T cells was significantly lower in tumor-involved lymph nodes compared to tumor-free ones (*P* = 0.025).

**Conclusions:**

Our data collectively indicate no significant differences in the frequencies of CD8^+^ lymphocytes or their memory subsets in tumor-draining lymph nodes of patients with BC. However, the frequency of CD45^low^ TCM was higher in tumor-involved nodes. Along with a decrease in the frequency of naive T cells, the higher frequency of CD45^low^ TCM suggests that despite the immune reaction to provide a pool of effective memory cells, it is blocked in early-stage of memory cells’ differentiation (CD45RO^low^), probably by tumor-derived suppressive factors. Identifying the molecular and cellular mechanisms behind this suppression can provide invaluable tools for adoptive T cell therapies in cancer.

## Background

Human immunological memory, a hallmark of the adaptive immune system, plays an important role in limiting the severity of infection and preventing morbidity [1]. For T lymphocytes, long-lasting immune protection is achieved by the differentiation of naive T cells upon antigen stimulation into distinct memory cell lineages: central (TCM) and terminally committed effector memory T cells (TEM). These cells are characterized by self-renewal capacity, clonal expansion, and faster attainment of effector functions upon antigen re-stimulation or challenge [2]. The repertoire of memory T cells was recently extended to diverse subtypes characterized by specific cell surface markers, unique homing properties, and special functional attributes [3].

T stem cells memory (TSCMs) were recently introduced as a rare subset of memory lymphocytes with the stem cell-like ability to self-renew and provide other memory and effector subsets [4–6]. Although these cells express naive markers they are more similar to memory subsets in function, as they express CD95 memory antigen and are antigen-experienced cells that respond rapidly to secrete effector cytokines [2]. These capabilities, along with recent evidence of their potential role in immune reconstitution in immunodeficient hosts and ability to mediate superior antitumor immunity in humanized mouse models, have brought TSCM cells to the attention of researchers in immunity and immunotherapies [7–9]. However, their role in tumor development and progression remains poorly understood [10].

In growing tumors, tumor-infiltrating lymphocytes have been shown to mediate an effective antitumor response. Among T cell subsets, CD8^+^ cells have been widely studied in cancer due to their ability to directly kill transformed cells [10, 11]. However, the suppressive tumor microenvironment often impairs their functionality through a set of transcriptional, functional, and phenotypic changes [10, 12]. Thus the present study, to extend our previous work on memory cells in tumors [13, 14], was designed to investigate the role of CD8^+^ lymphocytes and their memory cell subsets in tumor-draining lymph nodes (TDLNs) of patients with breast cancer (BC), and to identify their associations with clinical and pathological features.

## Methods

### Patients

Axillary lymph nodes (LNs) were obtained from 52 patients with BC who had undergone surgery for tumor resection. None of the patients had a history of chemotherapy or radiotherapy before surgery. A fresh part of each axillary LN was used for immunological assays, and the remaining tissue was used for routine pathological examination. Tumor infiltration into the nodes was determined histologically by pathologists. Nodes that were infiltrated by tumor cells were classified as node-positive (LN+). Patients were considered LN+ if at least one resected regional lymph node was observed to be infiltrated by tumor cells. Clinical and pathological information was obtained from the patients’ medical records. Their disease stage was determined with the TNM staging system according to the 7th edition of the AJCC cancer staging manual [15].

### Isolation of mononuclear cells from lymph nodes

To obtain a homogenous cell suspension, fresh LNs were mechanically minced into small pieces in complete culture medium [RPMI 1640 (Biosera, France)] containing 10% fetal bovine serum (FBS, Gibco, USA), 100 units/ml penicillin, and 100 μg/ml streptomycin (Biosera, France), and filtered through a 40-μm cell strainer (BD Biosciences, USA). Mononuclear cells were then isolated by centrifugation over a Ficoll-Hypaque density gradient (Biosera, France). The mononuclear ring was harvested and washed twice, and dissolved in 1 × phosphate-buffered saline (PBS) for further analysis. To determine the number of viable cells, the Trypan Blue dye (Biosera, France) exclusion test was used. Then cells at a concentration of 250 × 10^3^ in 50 μl 1 × PBS were distributed in round-bottomed polystyrene flow cytometry tubes (BD Biosciences, USA) for further analysis.

### Flow cytometry analysis

#### Antibodies

To determine the phenotype of memory T cell subsets, we used the following anti-human antibodies: FITC-anti-CCR7 (3D12), PE-anti-CD95 (Dx2), APC-conjugated anti-CD45RO (UCHL1), and PerCP anti-CD8 (Sk1), and their respective isotype controls: FITC-conjugated mouse IgG2a, PE-conjugated mouse IgG1, APC-conjugated mouse IgG2a, and PerCP-conjugated mouse IgG1 (all from BD Biosciences, USA).

### Cell staining

The mononuclear cells were surface-stained with appropriate fluorochrome-conjugated antibodies for 20 min at room temperature. The cells were then washed twice with 1 × PBS to remove unbound antibodies, and analyzed with a four-color FACSCalibur flow cytometer (BD Biosciences, USA).

### Flow cytometry data analysis

**Fig. 1:**
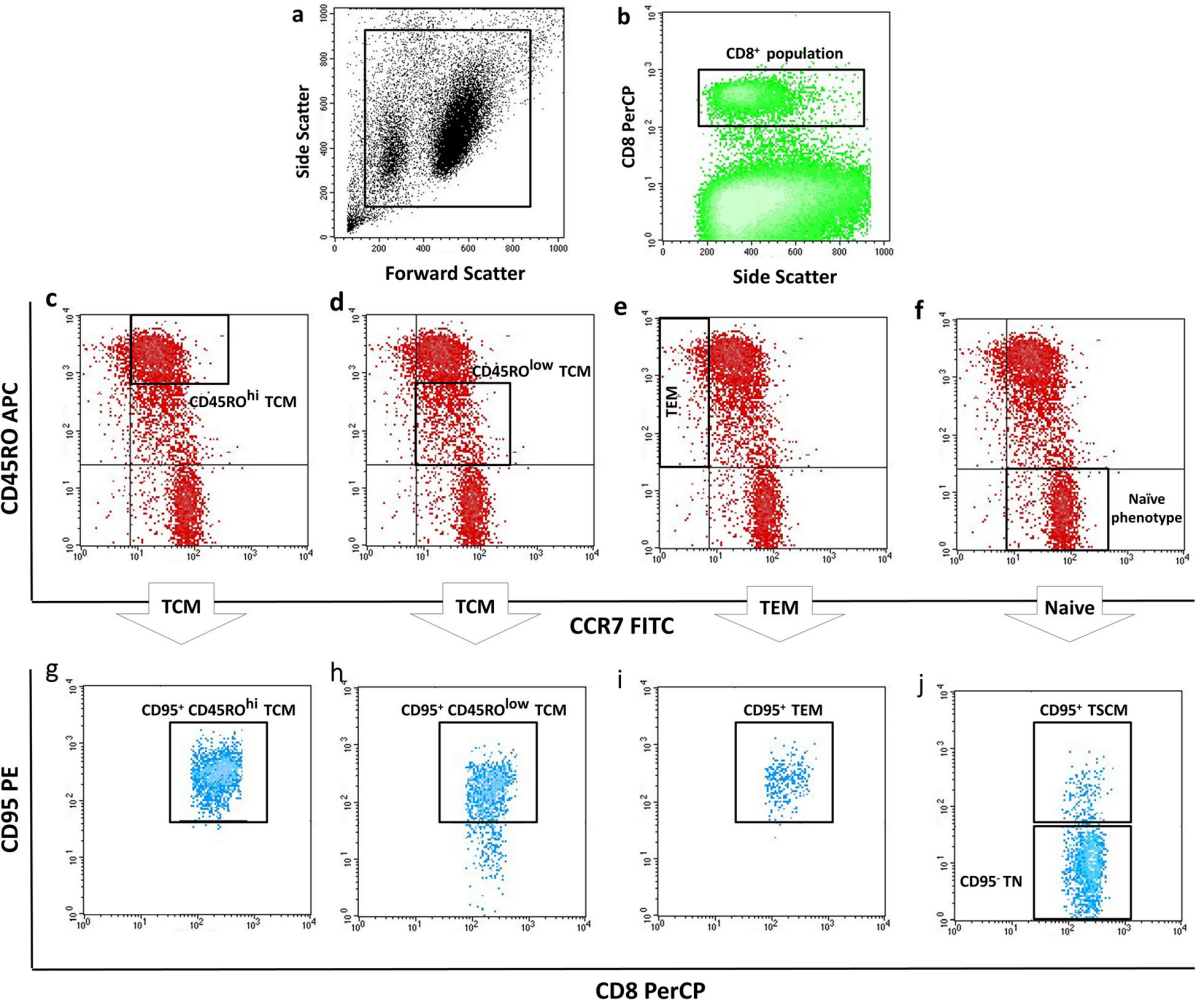
Phenotype determination of CD8^+^ memory T cell subsets in tumor-draining lymph nodes of patients with breast cancer. After selecting CD8^+^ positive cells in the lymphocyte gate (**a**), the phenotype of different subsets was defined based on the expression of CCR7, CD45RO and CD95. CD95 expressing CCR7^+^CD45RO^+^CD8^+^ lymphocytes were considered as TCM cells in both CD45RO^hi^ and CD45RO^low^ populations (**c, g** and **d, h**), whereas lymphocytes with a CCR7^−^CD45RO^+^CD95^+^CD8^+^ phenotype were considered as TEM cells (**e, i**) and CCR7^+^CD45RO^−^ cells that did not express CD95 were considered as naive cells (**f, j**). A subgroup of lymphocytes with naive phenotype (CCR7^+^CD45RO^−^) but positive for CD95 was coined as TSCM cells (**f, j**). TSCM: T memory stem; TCM: T central memory; TEM: T effector memory cells; TN: T naive

Flow cytometry data were analyzed with CellQuest Pro software (BD Biosciences, USA). Dead cells were first excluded according to their forward and side scatter (Fig. 1a). To determine the frequency of different memory T cell subsets, after selecting CD8^+^ cells in the lymphocytes gate (Fig. 1b), the phenotype of different subsets was defined based on the expression of CCR7, CD45RO, and CD95. Those CD95^+^CD8^+^ lymphocytes which expressed both CCR7 and CD45RO simultaneously, were considered TCM cells (Fig. 1c, d, g and h); the population with a CCR7^−^CD45RO^+^CD95^+^CD8^+^ phenotype was considered TEM cells (Fig. 1e and i); and CCR7^+^CD45RO^−^ cells that did not express CD95 were considered naive T cells (Fig. 1f and j). A subgroup of cells with the naive phenotype – CCR7^+^CD45RO^−^ – but positive for CD95 were considered TSCM cells (Fig. 1f and j). CD45RO expression on TCM cells was variable, so we divided these cells into CD45RO^hi^ TCM and CD45RO^low^ TCM subpopulations (Fig. 1c and d). Geometric mean florescence intensity (MFI) of CD95 was considered the criterion for expression level at the individual cell level. Each MFI was normalized to the MFI of unstained cells.

### Statistical analysis

The nonparametric Mann–Whitney U and Kruskal–Wallis H tests were used to identify statistically significant differences in subset frequencies between different patient subgroups. Correlations between the prevalence of each memory T cell subset and tumor size were determined by calculating Spearman’s rank correlation. SPSS 20 software (IBM Corp., Armonk, N.Y., USA) was used for all statistical analyses, and *P* values less than 0.05 (two-tailed) were considered significant. GraphPad Prism 6 software (GraphPad Software, Inc., USA) was used to draw the graphs.

## Results

After the diagnosis of BC was confirmed by pathological examination, 52 untreated patients with BC (mean age = 48.9 ± 1.55 years) were recruited into the study. According to the pathology reports, 23 out of 52 LNs were involved (44.23%). Most patients were in stage II (29/52, 55.77%), and in most, the tumor type was invasive ductal carcinoma (IDC, 41/50, 82.0%). The main clinical and pathological characteristics of the patients are summarized in Table 1.

**Table 1:** Clinical and pathological characteristics of patients with breast cancer

Characteristics	Value
**Age (years)**	48.9 ± 1.55
**Lymph node status**	
Free	29 (55.77%)
Involved	23 (44.23%)
N0	15 (28.85%)
N1	24 (46.15%)
N2	9 (17.31%)
N3	4 (7.69%)
**Stage**	
I	9 (17.31%)
II	29 (55.77%)
III	14 (26.92%)
**Tumor size**	
T1 (≤2)	20 (41.66%)
T2 (2–5)	28 (58.33%)
Unreported	4
**Tumor type**	
Invasive ductal carcinoma (IDC)	41 (82.00%)
Invasive lobular carcinoma (ILC)	3 (6.00%)
Invasive medullary carcinoma (IMC)	4 (8.00%)
Mixed IDC and ILC	2 (4.00%)
Unreported	2
**Histological grade**	
Well differentiated (I)	5 (11.63%)
Moderately differentiated (II)	27 (62.79%)
Poorly differentiated (III)	11 (25.58%)
Unreported	9
**Estrogen receptor (ER)**	
Negative	37 (82.22%)
Positive	8 (17.77%)
Unreported	7
**Progesterone receptor (PR)**	
Negative	11 (25.58%)
Positive	32 (74.42%)
Unreported	9
**Her2 expression**	
Negative	30 (58.82%)
Positive	15 (29.41%)
Equivocal	6 (11.76%)
Unreported	1
**Invasion**	
**Lymphatic invasion**	
Negative	11 (21.57%)
Positive	40 (78.43%)
Unreported	1
**Vascular invasion**	
Negative	11 (21.57%)
Positive	40 (78.43%)
Unreported	1
**Perineural invasion**	
Negative	5 (9.80%)
Positive	46 (90.20%)
Unreported	2

*All percentages are valid percent values. Missing data were excluded from the calculations

### Frequency of CD8^+^ memory T cell subsets in tumor-draining lymph nodes

The average frequency of different memory T cell subtypes in the CD8^+^ lymphocyte population along with mean expression of CD95 on the surface of these cells are reported in Table 2. As shown, 8.43 ± 0.49 of the lymphocytes in TDLNs of patients with BC were CD8-positive. In this group, more than 47% (47.65 ± 2.66%) of the cells expressed CD45RO, a marker of the memory T cell phenotype.

**Table 2:** Frequency of different memory CD8^+^ T cell subsets in tumor-draining lymph nodes of patients with breast cancer

Subset	Markers	Min	Max	Median	Mean ± SEM
**CD8**^**+**^**lymphocytes**	CD8^+^	2.7	18.19	7.57	8.43 ± 0.49
**CD45RO**^**+**^**CD8**^**+**^	CD8^+^CD45RO^+^	18.34	89.77	45.38	47.65 ± 2.66
**CD45RO**^**hi**^	CD8^+^CD45RO^hi^	5.58	66.50	28.38	29.10 ± 2.05
**CD45RO**^**low**^	CD8^+^CD45RO^low^	8.09	37.55	18.44	18.66 ± 0.89
**TCM**	CD8^+^CCR7^+^CD45RO^+^CD95^+^	8.91	75.12	31.42	33.84 ± 2.16
**CD45RO**^**hi**^	CD8^+^CCR7^+^CD45RO^hi^CD95^+^	3.97	57.80	20.21	22.49 ± 1.83
**CD45RO**^**low**^	CD8^+^CCR7^+^CD45RO^low^CD95^+^	3.0	30.28	11.89	13.61 ± 0.84
**CD8**^**+**^**TEM**	CD8^+^CCR7^−^CD45RO^+^CD95^+^	1.75	23.28	7.98	9.24 ± 0.78
**CD8**^**+**^**TSCM**	CD8^+^CCR7^+^CD45RO^−^CD95^+^	1.08	41.51	5.79	9.40 ± 1.37
**T Naive**	CD8^+^CCR7^+^CD45RO^−^CD95^−^	0.18	77.44	43.55	41.89 ± 2.89
**CD95**^**+**^**CD8**^**+**^	CD8^+^CD95^+^	15.82	91.35	54.93	54.90 ± 2.89
**CCR7**^**+**^**CD8**^**+**^	CD8^+^CCR7^+^	74.33	97.84	91.62	89.70 ± 0.82
**Mean expression of CD95 on different memory CD8**^**+**^**T cell subsets (based on MFI)**					
**TCM**	CD8^+^CCR7^+^CD45RO^+^CD95^+^	19.88	98.46	58.91	60.55 ± 2.90
**CD45RO**^**hi**^	CD8^+^CCR7^+^CD45RO^hi^CD95^+^	28.98	134.80	75.18	76.96 ± 3.58
**CD45RO**^**low**^	CD8^+^CCR7^+^CD45RO^low^CD95^+^	12.07	82.07	42.17	44.97 ± 2.12
**TEM**	CD8^+^CCR7^−^CD45RO^+^CD95^+^	30.35	121.92	67.48	71.43 ± 3.34
**TSCM**	CD8^+^CCR7^+^CD45RO^−^CD95^+^	5.61	84.95	22.89	27.80 ± 2.03

*TCM T central memory; *TEM* T effector memory; *TSCM* T stem cell memory; *TN* T naive

### Memory CD8^+^ T cell subsets in patients with different clinical and pathological characteristics

In the next step, we investigated the association of memory CD8^+^ subsets and naive CD8^+^ cells with different clinical and pathological parameters. Statistical analysis showed that the percentage of CD95^+^CD8^+^ and CD45RO^low^ CD8^+^ cells was significantly higher in involved lymph nodes comparing to tumor-free ones (*P* = 0.036 and *P* = 0.048, respectively). The percentage of CD45RO^+^CD8^+^ cells was also higher in patients with larger tumors (T2 vs. T1, *P* = 0.035). While the frequency of CD8^+^ lymphocytes was significantly lower in patients of N1 (with 1–3 involved nodes) and N2 (with 3–9 involved nodes) compared to patients with free nodes (*P* = 0.004 and *P* = 0.025, respectively).

### CD8^+^ TCM cells

**Fig. 2:**
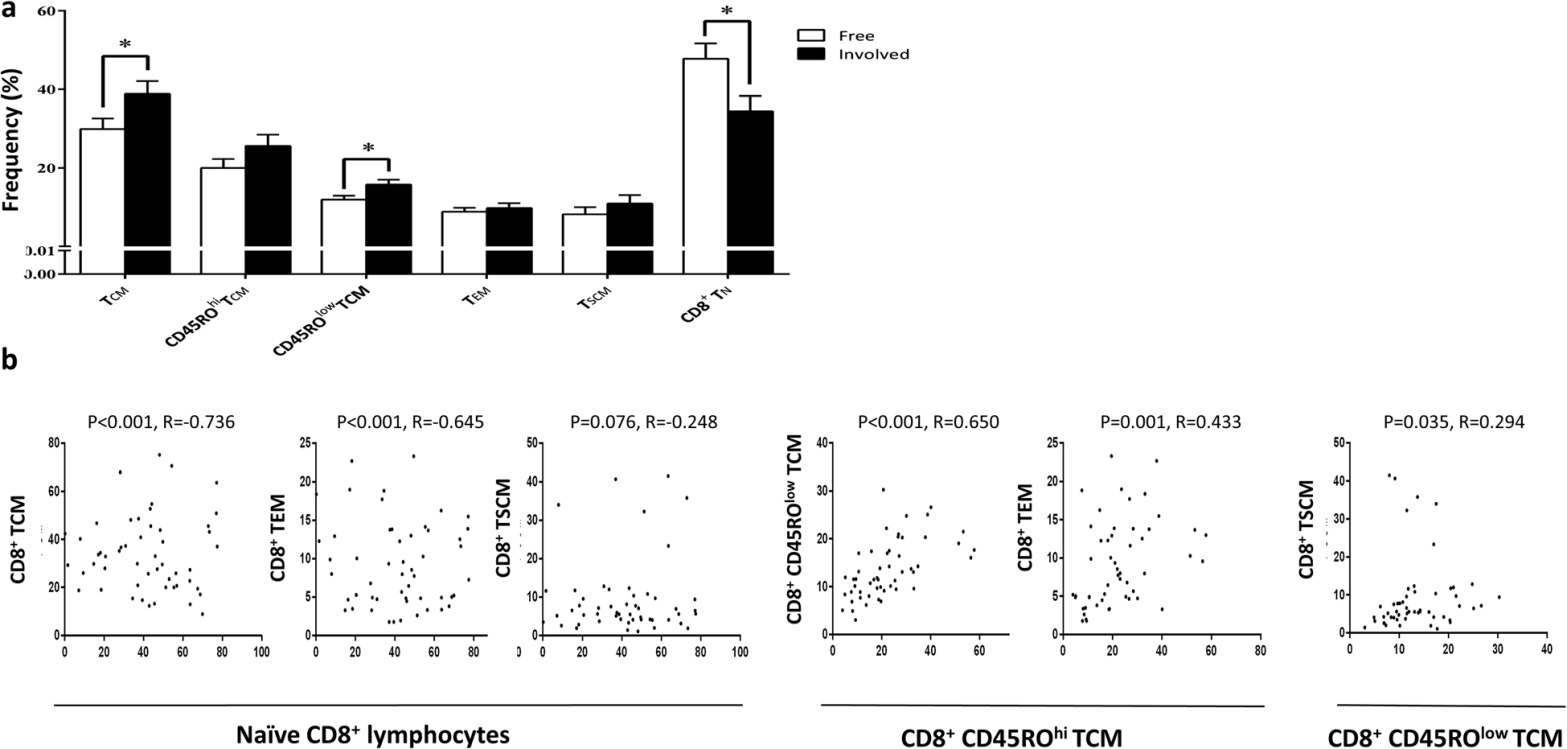
Frequency of memory cells in tumor-draining lymph nodes of patients with different nodal status as well as their correlation. The percentages of different CD8^+^ memory cell subsets in draining lymph nodes of breast cancer patients with different statuses of lymph nodes involvement. (**a**) The frequency of TCM and their CD45RO^low^ subset were significantly higher in tumor-involved lymph nodes. Part (**b**) shows significant correlations among different CD8^+^ lymphocyte subsets. Data are presented as the mean ± SEM. *Significant difference at the 0.05 level (two-tailed)

The total frequency of TCM with the CD8^+^CCR7^+^CD45RO^+^CD95^+^ phenotype was 33.84 ± 2.16 in draining lymph nodes of patients with BC. We also investigated two different subsets with low and high CD45RO expression (Table 2). Our analysis showed that the frequency of total TCM cells and the subset with low CD45RO expression (CD45RO^low^ TCM) was significantly greater in involved nodes compared to tumor-free ones (*P* = 0.024 and *P* = 0.017, respectively; Fig. 2a). Among patients in different pathological stages, CD95 expression (based on MFI) on the surface of TCM overall, and the CD45RO^hi^ TCM and CD45RO^low^ TCM subsets, was higher only in patients with stage II compared to those with stage I disease (*P* = 0.004, *P* = 0.015 and *P* = 0.001, respectively). In addition, the expression of CD95 on TCM was higher in TDLNs of patients with moderately differentiated tumor cells (grade II) compared with those with well-differentiated tumors (grade I, *P* = 0.019). Regarding the number of involved lymph nodes, CD95 expression on TCM, CD45RO^hi^ and CD45RO^low^ TCM subsets was notably higher in patients with N1 disease compared to the node-free group (*P* < 0.0001, *P* = 0.002 and *P* < 0.001, respectively). Mean fluorescence intensity for CD95 on TCM cells was also greater in the N3 group (*P* = 0.037) compared to the N0 group. Furthermore, the percentage of CD45RO^hi^ TCM cells was also greater in patients with larger tumor sizes (T2 vs. T1; *P* = 0.038).

### CD8^+^ TEM cells

Approximately 9% of CD8^+^ cells (9.24 ± 0.78%) in draining LNs of patients with BC had the effector memory phenotype (CD8^+^CCR7^−^CD45RO^+^CD95^+^). Analysis of CD95 expression on TEM cells in patients with different clinical and pathological characteristics indicated higher expression of this molecule in N1 patients compared to node-free patients (*P* = 0.020).

### CD8^+^ TSCM cells

The frequency of the CD8^+^CCR7^+^CD45RO^−^CD95^+^ phenotype, considered here to reflect TSCM cells, was 9.40 ± 1.37%. Although the frequencies of these cells did not differ significantly among patients with different clinical and pathological characteristics, mean expression of CD95 (based on MFI) on the surface of TSCM showed an increase in patients with stage II (*P* = 0.012) compared to those in stage I. The expression of CD95 on these cells was also greater in TDLNs of N1 patients compared to node-free patients (N0, *P* = 0.003).

### Naive CD8^+^ lymphocytes

In addition to memory CD8^+^ lymphocytes, we also determined the percentage of lymphocytes with the naive phenotype (CCR7^+^CD45RO^−^CD95^−^) in TDLNs. The percentage of naive CD8^+^ T cells was significantly lower in tumor-involved lymph nodes compared to tumor-free ones (*P* = 0.022, Fig. 2a).

### Correlations among frequencies of different CD8^+^ lymphocyte subsets

We also investigated the correlations among different subsets, and between subsets and patients’ age, with the Spearman correlation test (Fig. 2b). The results showed that the percentage of naive cells had a strong negative correlation with TCM (*P* < 0.001, R = − 0.736), their CD45RO^hi^ (P < 0.001, R = − 0.773) and CD45RO^low^ (P < 0.001, R = − 0.682) subsets, and TEM cells (P < 0.001, R = − 0.645). The percentage of CD45RO^low^ TCM cells had a positive correlation with CD45RO^hi^ TCM (P < 0.001, R = 0.650) and TSCM (*P* = 0.035, R = 0.294) subsets, and the percentage of CD45RO^hi^ TCM cells correlated strongly with TEM cells (*P* = 0.001, R = 0.433). We also observed a positive association between age and the frequency of CD45RO^+^CD8^+^ (*P* = 0.042, R = 0.283), TCM (*P* = 0.022, R = 0.318) and TSCM (*P* = 0.049, R = 0.275) subsets. Conversely, a negative correlation was observed between age and the frequency of TN (*P* = 0.025, R = − 0.0310).

## Discussion

To our knowledge the present study is the first to investigate the presence and associations of CD8^+^ memory T cell subsets in TDLNs from patients with BC. On average, 8% of lymphocytes were positive for CD8, representing cytotoxic lymphocytes; almost half of them expressed the CD45RO memory cell marker (Table 2). The frequency of these memory cells did not differ among patients with different clinical and pathological characteristics; however, the frequencies of memory subpopulations, TCM and its subset with low CD45RO expression were significantly higher in tumor-involved lymph nodes.

There is a general consensus that in the context of the antitumor immune response, the frequency of CD8^+^ lymphocytes and their memory T cell subsets correlates positively with smaller tumor size, lower disease stages, less lymph node involvement, and a generally better prognosis or survival in most types of cancer, e.g. breast carcinoma [14, 16–22]. CD8^+^ lymphocytes are assumed to mediate tumor rejection through the direct killing of transformed cells. Consistently, we observed a reduction in the frequency of total CD8^+^ lymphocytes and their naive subset along with tumor dissemination to draining lymph nodes. On the other hand, an increased frequency of CD45RO^+^CD8^+^ lymphocytes was shown to be associated with larger tumor size. Similar results were obtained in our previous study regarding some CD4^+^ memory subsets [13]. In line with our observation, Feuerer and colleagues also found that the number of memory cells (CD4^+^/CD8^+^ CD45RO^+^) in the bone marrow of patients with BC increased in parallel with tumor cell metastases to the bone marrow [23, 24]. This may simply reflect an immune system attempt to provide an antitumor immune response after encountering antigens.

It is now well documented that the CD45RO marker cannot unequivocally define the memory T cell phenotype, since other effectors such as B and NK cell subsets also express CD45RO. In addition, different subtypes of memory cells have different functionality and homing properties. Despite the well-known role of memory cells in the defense against tumors, the role of their subsets has been rarely studied in cancer. Hence, we also investigated different subpopulations of CD8^+^ memory T cells in TDLNs of patients with BC.

Memory CD8^+^ T cells are conventionally divided to two main subsets: TCM and TEM. While TCM cells express the CCR7 homing receptor and show less differentiation, higher self-renewal potential and increased proliferation, TEM cells are commonly characterized by a phenotype more similar to that of effector cells, i.e. high cytotoxicity, rapid effector function and high IFNγ secretion [25, 26]. We observed that more than 33% of CD8^+^ lymphocytes in TDLNs of patients with BC had the TCM phenotype, versus 9% of cells with the TEM phenotype. The lower frequency of TEM cells is consistent with the migration of these cells to inflammatory sites, as also observed for CD4^+^ TEM in our previous study [13]. A predominant frequency of TEM cells in tumor microenvironments has been reported in different murine and human tumor models [25]. In our patients there were no remarkable differences in the percentages of TEM cells in relation with different clinical and pathological characteristics; however, we observed that the total percentage of TCM cells and their CD45RO^low^ subset was much higher in involved nodes compared to tumor-free ones. Similar results were reported for CD4^+^ lymphocytes in our previous study of CD4^+^ TCM subsets [13]. Considering the fact that CD45RO expression increases in parallel with differentiation in memory cells, these findings along with the increased expression of CD95 on TCM and their subsets in patients with advanced tumors (i.e. higher stage, higher grade, and more tumor-involved nodes) suggest that in BC, although the immune system tries to provide a pool of effective memory cells against the tumor, interactions with, or signals from, transformed cells and the tumor microenvironment lead to changes that diminish the host’s ability to eradicate the tumor. Concordant with this hypothesis, a number of earlier studies found that in growing tumors, immune cells in the tumor milieu, draining lymph nodes and peripheral blood from patients with BC are often functionally impaired or have regulatory phenotypes [27–31]. In addition to the fact that the CD45RO marker is also highly expressed on regulatory T cells [32], it has also been shown that by regulating the threshold of sensitivity, CD45 expression regulates cellular responses [33]. Accordingly, the failure in tumor immunological responses appears to be partly due to the suppression of memory cell differentiation or function as the disease progresses.

Our findings also provide the first evidence, to our knowledge, of the presence of a new subset of memory CD8^+^ cells, TSCM, in TDLNs of patients with BC. These cells are known to be highly proliferative, self-renewing, and multipotent, and to have the potential ability to differentiate into other memory subsets; accordingly, they have been named memory stem cells. In the present study, TSCM cells (CD8^+^CCR7^+^CD45RO^−^CD95^+^) represented more than 9% of CD8^+^ cells; however, the frequency of these cells did not differ significantly in women with different clinical and pathological characteristics. Nevertheless, we observed that mean expression of CD95 on the surface of TSCM was higher in patients with higher-stage BC and lymph node involvement. Regarding CD4^+^ TSCM, we observed that they were more frequent in tumor-involved lymph nodes and in patients with advanced-stage disease [13]. Few studies to date have aimed to investigate the role of TSCM in cancer, but some recent work, focused on the distribution and function of TSCM in antitumor immune responses, showed an increased frequency of CD4^+^ and CD8^+^ TSCM cells in blood and lymph nodes from patients with non-small-cell lung cancer [34], and in patients with acute-phase adult T cell leukemia, in which TSCM are considered to be a reservoir for the HTLV-1 virus [35].

## Conclusion

Our data constitute evidence that the frequencies of CD8^+^ lymphocytes as well as their TSCM subsets do not change significantly in draining lymph nodes of patients with BC. However, the frequency of CD8^+^ memory subsets with low CD45RO expression was higher in tumor-affected nodes. These observations provide some support for our previous hypothesis that in BC, following constant, long-term exposure to tumor antigens, the patient’s immune system attempts to provide a pool of effective memory cells. Nevertheless, tumor-derived suppressive factors appear to block memory cell differentiation in the early stages of the disease (CD45RO^low^). Identifying the molecular and cellular mechanisms behind this suppression holds the potential to provide invaluable tools for adoptive T cell therapies in cancer.
